# The association of urinary heavy metal exposure with frailty susceptibility and mortality in middle-aged and older adults: a population-based study

**DOI:** 10.1186/s13690-024-01275-8

**Published:** 2024-03-27

**Authors:** Zitian Zheng, Huanhuan Luo, Qingyun Xue

**Affiliations:** 1 Department of Orthopedics, Beijing Hospital, National Center of Gerontology, Institute of Geriatric Medicine, Chinese Academy of Medical Sciences, NO.1 Da Hua Road, 100730 DongDan, Beijing, P.R. China; 2 Department of Sports Medicine, Beijing Key Laboratory of Sports Injuries, Peking University Third Hospital, Institute of Sports Medicine of Peking University, Beijing, P.R. China; 3 Engineering Research Center of Sports Trauma Treatment Technology and Devices, Ministry of Education, Beijing, P.R. China; 4 Peking University Fifth School of Clinical Medicine, Beijing, P.R. China; 5 Department of Nursing, Beijing Hospital, National Center of Gerontology, Institute of Geriatric Medicine, Chinese Academy of Medical Science, Beijing, P.R. China; 6 Graduate School of Peking Union Medical College, Beijing, P.R. China

**Keywords:** Chemical mixture, Frailty, Heavy metal, Weighted quantile sum regression, Bayesian kernel machine regression

## Abstract

Heavy metals’ presence as environmental pollutants has a close link to adverse health effects. Frailty, a clinical syndrome hallmarked by elevated vulnerability to stressors, presents a substantial challenge in healthcare. However, the association between exposure to heavy metals and frailty largely remains unexplored. Utilizing data from the National Health and Nutrition Examination Survey (NHANES) spanning 2003–2018 and correlated with the U.S. National Death Index (NDI) from 2019, we investigated mortality outcomes. Logistic regression, Cox regression, Kaplan-Meier survival curves, weighted quantile-sum (WQS) regression, and Bayesian kernel machine regression (BKMR) were employed to assess the association between heavy metal exposure and frailty incidence and mortality in the frail population. Eight metals were measured in urine using inductively coupled plasma mass spectrometry with values adjusted for urinary creatinine, which was used to reflect heavy metal exposure. The cohort incorporated 5370 female participants aged 45 and above, with 1518 diagnosed with frailty. The findings indicated a substantial correlation between exposure to specific heavy metals, namely tungsten (odds ratio [OR]: 1.94, 95% confidence interval [CI]: 1.31–2.89), cobalt (OR: 1.64, 95% CI: 1.40–1.93), cadmium (OR: 1.93, 95% CI: 1.52–2.43), and uranium (OR: 7.36, 95% CI: 1.53–35.28), and an elevated risk of frailty. WQS and BKMR regression models identified cadmium, cobalt, and tungsten as main contributors to frailty. Cox regression analysis, after adjustment for covariates, suggested that the higher the exposure levels to cadmium and lead, the higher the risk of death in frail patients, with associated hazard ratios (HR) of 95% CI: 1.96 (1.53, 2.52) and 1.30 (1.13, 1.49), respectively. Our study revealed a significant positive correlation between exposure to heavy metal mixtures and frailty onset in middle-aged and older adults, along with increased mortality in frail patients. Cobalt, cadmium, and tungsten emerged as prominent contributors to frailty, with cobalt and cadmium directly impacting the long-term life expectancy of frail patients.

**Supplementary Information:**

The online version contains supplementary material available at 10.1186/s13690-024-01275-8.

**Table:** 

Text box 1. Contributions to the literature	
• The study deepens our understanding of the link between heavy metal exposure and frailty, an underexplored area in public health, highlighting the need for integrating environmental and clinical factors in health assessments. • By employing a mix of traditional and advanced statistical models, this research advances the methodological toolkit available for studying the health impacts of environmental pollutants. • This study identifies cadmium, cobalt and tungsten exposure as independent contributors to frailty in the middle-aged and older population. • This study identifies cadmium and cobalt as independent risk factors for increased mortality in frail populations.	

## Introduction

The progressive advancement in human longevity has precipitated burgeoning interest in frailty, a multidimensional syndrome inextricably linked to aging [1]. Frailty is emblematic of the declining capacity of an individual to recuperate from illnesses or external stressors [2], signifying a gradual deterioration of various physiological systems and the consequent disruption to homeostasis [3]. Intriguingly, individuals within the same age cohort may exhibit significant discrepancies in their baseline health status and increased susceptibility to adverse outcomes [4]. This differential vulnerability is broadly hypothesized to stem from varying levels of frailty [5]. Although frailty can manifest at any life stage, its prevalence escalates in the older demographics [6], thereby rendering it a critical global public health concern [7]. Consequently, there is an urgent need to identify and verify the modifiable risk factors correlated with frailty.

In response to the rapid pace of global industrialization, the health implications of heavy metal exposure are escalating [8]. A substantial body of empirical evidence derived from numerous studies has underscored the varied effects of heavy metal exposure on human health [9]. These effects encompass an array of conditions including increased risk of cardiovascular disease [10], hepatic and renal pathologies [11], and musculoskeletal disorders [12]. Moreover, these accruing physical impairments further reduce the body’s resistance to external stresses, potentially heightening the risk of frailty [13], which in turn may precipitate a further decline in physical function and disease [14]. The positive association between exposure to phthalates and polycyclic aromatic hydrocarbons with frailty has been elucidated [15]. Nevertheless, the nature of the association between frailty and heavy metal exposure remains poorly understood. Current research efforts primarily focus on the repercussions of singular exposure to heavy metals, particularly focusing on lead and cadmium, and their influence on frailty incidence [16, 17]. Such studies often overlook the intricate effects of concurrent exposure to a cocktail of heavy metals [18], leading to a partial comprehension of the impact of heavy metal exposure on population health and disease trajectory [19]. Accordingly, the present gaps in research warrant addressing through the use of more sophisticated statistical methodologies to explore the interdependence between mixtures of heavy metals and frailty incidence [20]. Importantly, individuals in vulnerable groups are more susceptible to external stressors, such as heavy metal exposure, and to the detrimental consequences arising from trauma [21]. Yet, the degree to which heavy metals can directly increase patient mortality remains enveloped in uncertainty. Our study seeks to further explore the relationship between heavy metal exposure and mortality among frail patients.

Our research is premised on the hypothesis of a correlation between both the incidence and mortality of frailty and heavy metal exposure. We additionally aim to identify which constituents in mixtures of heavy metals exert the most significant influence on frailty. Our study utilizes data acquired from eight cycles of the National Health and Nutrition Examination Survey (NHANES) spanning from 2003 to 2018 [22]. We employ multipollutant analytical approaches, harnessing both weighted quantile sum (WQS) regression [23] and Bayesian kernel machine regression (BKMR) [24]. Given that frail individuals exhibit a heightened propensity for severe, life-endangering complications in response to external stressors - ultimately escalating mortality, we have further applied Cox regression and Kaplan Meier (KM) survival curve methodologies to analyze the relationship between heavy metals and mortality [25].

## Methods

### Study population

We utilized data from eight cycles of the US National Health and Nutrition Examination Survey (NHANES) conducted from 2003 to 2018. The National Center for Health Statistics (NCHS) Research Ethics Committee granted ethical approval for the NHANES protocol and methodology, and all participants provided written informed consent. Given that our analysis was based on publicly accessible data for exploratory purposes, additional approval from a local Ethics Committee was not required. A total of 80,312 participants were initially enrolled. We excluded participants who were younger than 45 years of age, or had incomplete information on the eight trace metals, or had incomplete covariates, or had missing follow-up data, resulting in a final sample size of 5370 participants. For more details on the participant screening process, see Supplementary Figure S1.

### Frailty index criteria

Frailty was assessed using the standardized methodology developed by Searle et al. [26]. The frailty index incorporated 49 deficits across various domains, including cognition, dependence, depression, and comorbidities, among others. Each health deficit received a severity score ranging from 0 to 1, based on its respective impact. The frailty index value was computed by dividing the accumulated deficits by the total number of potential deficits. Participants were categorized into two groups: the frailty group (frailty index value of 0.25 or higher) and the non-frailty group (frailty index value below 0.25). For a comprehensive overview of the variables included in the frailty index and their corresponding scores, please refer to Supplementary Table S1 [27].

### Mortality

Information on mortality status and follow-up time was obtained through the National Death Index mortality database associated with NHANES (up to April 26, 2022). The cause of death was ascertained using the International Classification of Diseases 10th Revision, and the follow-up outcomes of our study were all-cause mortality (I00–I09, I11, I13, I20–I51).

### Metal measurement

The study utilized data on eight urinary metals (cadmium, cobalt, cesium, molybdenum, antimony, thallium, tungsten, and uranium) obtained from the NHANES surveys conducted between 2003 and 2018. These metals were measured in spot urine samples using inductively coupled plasma mass spectrometry (ICP-MS). For values falling below the limit of detection (LOD), the standard practice of substituting them with the LOD divided by the square root of two was applied [28]. The limits of detection (LODs) for the eight urinary metals were presented in Supplementary Table S2, and the detection rates were all greater than 70%. While the NHANES database does not transform data that are above the LOD but below the limit of quantification (LOQ) nor reports the LOQ for the corresponding test method, it is pertinent to mention that NHANES employs extremely rigorous laboratory measurement practices, ensuring the highest standards of data reliability and validity [29, 30]. Furthermore, in precedent high-quality literature utilizing NHANES data [31, 32], no special transformations or treatments were applied to this data [33]. And given the large sample size of this study, it is believed that this will not significantly impact our results and conclusions. All urinary metals were adjusted for urine dilution using individual urinary creatinine, and reported as micrograms of metal per gram of creatinine [32].

### Covariate ascertainment

To enhance analysis accuracy and reliability, we employed various covariates including age, sex, race, poverty income ratio (PIR), educational level, body mass index (BMI), alcohol intake, smoking status, hypertension, diabetes, serum cotinine, physical activity, and estimated glomerular filtration rate (eGFR). eGFR was determined using the Chronic Kidney Disease Epidemiology Collaboration equation [34], with participants grouped into normal renal function (eGFR > 90 mL/min/1.73 m²), modestly declined renal function (eGFR: 60–90 mL/min/1.73 m²), and CKD group (eGFR < 60 mL/min/1.73 m²) [35]. Physical activity was quantified via an interview questionnaire, with energy expenditures classified based on Ainsworth’s criteria [36]. The subjects were classified into different groups based on their PA level. The groups were defined as Very Low PA (VLPA) (< 150 MET-min/week), Low PA (LPA) (150–960 MET-min/week), Medium PA (MPA) (961–1800 MET-min/week) and High PA (HPA) (> 1800 MET-min/week) [37].

### Statistical analysis

Participants’ demographic characteristics, stratified by frailty status, were examined using descriptive statistics, t-tests, and Mann-Whitney U tests. Urinary creatinine-standardized heavy metal concentrations were log2 transformed to approximate normal distribution. Logistic regression models were used to investigate links between heavy metals and frailty risk, reported as odds ratios (OR) and 95% confidence intervals (CI). Cox proportional risk regression models estimated hazard ratios (HRs) and 95% CIs for all-cause mortality associated with urinary heavy metal concentrations as both continuous and categorical variables.

To assess cumulative metal impacts on frailty and construct a metal mixture index, we employed a combination of statistical methods, including the weighted quantile sum (WQS) regression via the “gWQS” R package [38]. Robust parameter estimation was assured through 5000 bootstrap replications.

The Bayesian kernel machine regression (BKMR) model was employed to assess cumulative urinary heavy metal impacts on frailty risk, accounting for potential non-linear and non-additive relationships within the exposure mixtures [24]. Each heavy metal’s influence on frailty prevalence was evaluated via the posterior inclusion probability (PIP), with a 0.5 threshold indicating significance. The BKMR model incorporates both Bayesian and statistical learning techniques, allowing flexible modeling of exposure-response functions and visualization of effects from individual or combined exposures.

Values of *p* < 0.05 were considered significant. All statistical analyses were conducted using R statistical software (version 4.2.0).

## Results

### Characteristics of participants and metals distribution

In this research, a total of 5370 adults aged 45 years or above took part, among whom 1518 individuals were diagnosed with frailty. Table 1 presents the demographic characteristics of the study participants, both with and without frailty. Several variables, such as age, gender, ethnicity, family income-poverty ratio, BMI, education level, smoking status, alcohol intake status, hypertension status, diabetes status, physical activity and eGFR, exhibited statistically significant differences between the frail and non-frail groups.

**Table 1:** Characteristics of study participants by frailty status

Characteristics	Total population (*n* = 5370)	Non-frailty (*n* = 3852)	Frailty (*n* = 1518)	*P*-value
Age, n(%)				< 0.001
< 65	3110 (57.91%)	2353 (61.09%)	757 (49.87%)	
≥ 65	2260 (42.09%)	1499 (38.91%)	761 (50.13%)	
Gender, n(%)				< 0.001
Male	2717 (50.60%)	2032 (52.75%)	685 (45.13%)	
Female	2653 (49.40%)	1820 (47.25%)	833(54.87%)	
Race/ethnicity, n(%)				< 0.001
Mexican American	1132 (21.08%)	913 (23.70%)	219 (14.43%)	
Other Hispanic	546 (10.17%)	361 (9.37%)	185 (12.19%)	
Non-Hispanic White	1838 (34.23%)	1280 (33.23%)	558 (36.76%)	
Non-Hispanic Black	1466 (27.30%)	1063 (27.60%)	403 (26.55%)	
Other race including Multi-Racial	388 (7.23%)	235 (6.10%)	153 (10.08%)	
Family income-poverty ratio, n (%)				< 0.001
<1.3	917 (17.08%)	593 (13.31%)	474 (26.50%)	
1.3–2.4	2337 (43.52%)	1836 (41.22%)	882 (49.33%)	
≥2.4	2116 (39.40%)	2025 (45.46%)	433 (24.20%)	
Body mass index, n(%)	29.26 ± 6.40	28.56 ± 5.74	31.02 ± 7.54	< 0.001
Higher education, n (%)				< 0.001
No	2801 (52.16%)	1859 (48.26%)	942 (62.06%)	
Yes	2569 (47.84%)	1993 (51.74%)	576 (37.94%)	
Smoking status, n (%)				< 0.001
Never	2610 (48.60%)	1978 (51.35%)	632 (41.63%)	
Ever	1985 (36.96%)	1366 (35.46%)	619 (40.78%)	
Now	775 (14.43%)	508 (13.19%)	267 (17.59%)	
Alcohol intake, n (%)				0.006
No	3682 (68.57%)	2685 (69.70%)	997 (65.68%)	
Yes	1688 (31.34%)	1167 (30.30%)	521 (34.32%)	
Hypertension, n(%)				0.003
No	3819 (71.12%)	2784 (72.27%)	1035 (68.18%)	
Yes	1551 (28.88%)	1068 (27.73%)	483 (31.82%)	
Diabetes, n(%)				< 0.001
Yes	4204 (78.29%)	3317 (86.11%)	887 (58.43%)	
No	991 (18.45%)	427 (11.09%)	564 (37.15%)	
Borderline	175 (3.26%)	108 (2.80%)	67 (4.41%)	
Physical Activity, n(%)				< 0.001
Very Light Physical Activity	1709 (31.82%)	999 (25.93%)	710 (46.77%)	
Light Physical Activity	1330 (24.77%)	955 (24.79%)	375 (24.70%)	
Medium Physical Activity	668 (12.44%)	530 (13.76%)	138 (9.09%)	
High Physical Activity	1663 (30.97%)	1368 (35.51%)	295 (19.43%)	
Serum cotinine (ng/mL), mean ± SD	54.19 ± 134.16	48.68 ± 126.99	65.70 ± 145.54	< 0.001
eGFR(mL/min/m), n(%)				< 0.001
≥ 90	1592 (29.79%)	1235 (32.22%)	357 (23.63%)	
60–90	2835 (52.79%)	2109 (54.75%)	726 (47.83%)	
< 60	943 (17.65%)	508 (13.25%)	435 (28.79%)	

VLPA: Very Light Physical Activity (< 150 MET-min/week); LPA: Light Physical Activity (150–960 MET-min/week); MPA: Medium Physical Activity (961–1800 MET-min/week); HPA: High Physical Activity (> 1800 MET-min/week); eGFR: estimated glomerular filtration rate

### Logistic regression to analyze the associations of individual metals with frailty

The results from the logistic regression models, as shown in Table 2, demonstrate significant associations between log-transformed metal concentrations and the risk of frailty. Tungsten (OR 1.96, 95% CI 1.32 to 2.92), cadmium (OR 1.90, 95% CI 1.51 to 2.40), cobalt (OR 1.64, 95% CI 1.40 to 1.93), and uranium (OR 7.84, 95% CI 1.63 to 37.59) display positive associations, indicating an increased risk of frailty. These findings remain consistent even after adjusting for all covariates in the fully covariate-adjusted model, with a P value for trend less than 0.05 when transforming the variables into categorical variables. On the other hand, none of the metals showed a statistically significant negative correlation with frailty risk.

**Table 2:** OR (95% CI) in frailty associated with log-urinary heavy metals levels according to multivariate logistic regression

Urine Metals (log-µg/g creatinine)	Model 1		Model 2		Model 3	
	OR (95% CI)	*P* value	OR (95% CI)	*P* value	OR (95% CI)	*P* value
**Cadmium**						
Continuous	2.13 (1.79, 2.55)	< 0.01	1.55 (1.29, 1.87)	< 0.01	1.90 (1.51, 2.40)	< 0.01
Q1	Reference		Reference		Reference	
Q2	1.28 (1.07, 1.52)	0.01	1.15 (0.96, 1.39)	0.13	1.23 (1.01, 1.51)	0.04
Q3	1.40 (1.18, 1.67)	< 0.01	1.16 (0.97, 1.39)	0.11	1.25 (1.01, 1.54)	0.04
Q4	1.97 (1.66, 2.34)	< 0.01	1.47 (1.23, 1.76)	< 0.01	1.71 (1.37, 2.14)	< 0.01
***P*** **for trend**	2.54 (2.02, 3.19)	< 0.01	1.69 (1.33, 2.16)	< 0.01	2.06 (1.53, 2.78)	< 0.01
**Cobalt**						
Continuous	1.49 (1.29, 1.71)	< 0.01	1.39 (1.20, 1.61)	< 0.01	1.64 (1.40, 1.93)	< 0.01
Q1	Reference		Reference		Reference	
Q2	0.89 (0.75, 1.05)	0.17	0.84 (0.70, 1.00)	0.05	0.99 (0.81, 1.20)	0.91
Q3	1.04 (0.87, 1.23)	0.69	0.94 (0.79, 1.13)	0.52	1.22 (1.00, 1.49)	0.05
Q4	1.42 (1.21, 1.68)	< 0.01	1.25 (1.05, 1.49)	0.01	1.77 (1.45, 2.15)	< 0.01
***P*** **for trend**	2.12 (1.60, 2.81)	< 0.01	1.73 (1.28, 2.34)	< 0.01	3.02 (2.16, 4.22)	< 0.01
**Cesium**						
Continuous	0.73 (0.66, 0.81)	< 0.01	0.73 (0.65, 0.81)	< 0.01	0.93 (0.82, 1.04)	0.20
Q1	Reference		Reference		Reference	
Q2	0.81 (0.69, 0.95)	0.01	0.84 (0.70, 0.99)	0.04	1.03 (0.86, 1.25)	0.72
Q3	0.68 (0.58, 0.80)	< 0.01	0.69 (0.58, 0.82)	< 0.01	0.89 (0.74, 1.08)	0.25
Q4	0.63 (0.53, 0.74)	< 0.01	0.62 (0.51, 0.74)	< 0.01	0.89 (0.73, 1.10)	0.28
***P*** **for trend**	0.69 (0.61, 0.78)	< 0.01	0.68 (0.60, 0.78)	< 0.01	0.90 (0.77, 1.04)	0.16
**Molybdenum**						
Continuous	1.01 (0.95, 1.08)	0.71	0.97 (0.90, 1.03)	0.29	1.04 (0.97, 1.12)	0.31
Q1	Reference		Reference		Reference	
Q2	1.00 (0.84, 1.18)	0.97	0.91 (0.77, 1.09)	0.32	0.97 (0.80, 1.17)	0.72
Q3	1.05 (0.88, 1.24)	0.60	0.94 (0.79, 1.13)	0.52	1.03 (0.85, 1.24)	0.79
Q4	1.14 (0.96, 1.34)	0.14	0.99 (0.83, 1.18)	0.89	1.18 (0.97, 1.43)	0.10
***P*** **for trend**	1.07 (0.99, 1.16)	0.11	1.00 (0.92, 1.09)	0.99	1.09 (0.99, 1.19)	0.08
**Antimony**						
Continuous	1.22 (0.71, 2.09)	0.48	0.89 (0.50, 1.59)	0.70	1.13 (0.60, 2.12)	0.70
Q1	Reference		Reference		Reference	
Q2	1.11 (0.94, 1.32)	0.23	1.03 (0.86, 1.23)	0.74	1.06 (0.88, 1.29)	0.53
Q3	1.27 (1.07, 1.50)	0.01	1.17 (0.98, 1.40)	0.08	1.26 (1.04, 1.53)	0.02
Q4	1.39 (1.18, 1.65)	< 0.01	1.22 (1.03, 1.46)	0.03	1.39 (1.15, 1.69)	< 0.01
***P*** **for trend**	9.79 (3.14, 30.56)	< 0.01	4.38 (1.33, 14.46)	0.02	10.95 (2.96, 40.44)	< 0.01
**Thallium**						
Continuous	0.20 (0.12, 0.33)	< 0.01	0.24 (0.14, 0.40)	< 0.01	0.58 (0.34, 1.01)	0.06
Q1	Reference		Reference		Reference	
Q2	0.61 (0.51, 0.71)	< 0.01	0.61 (0.52, 0.73)	< 0.01	0.70 (0.59, 0.85)	< 0.01
Q3	0.54 (0.46, 0.64)	< 0.01	0.56 (0.47, 0.67)	< 0.01	0.69 (0.57, 0.83)	< 0.01
Q4	0.50 (0.42, 0.59)	< 0.01	0.53 (0.44, 0.63)	< 0.01	0.72 (0.59, 0.88)	< 0.01
***P*** **for trend**	0.08 (0.04, 0.15)	< 0.01	0.10 (0.05, 0.20)	< 0.01	0.33 (0.15, 0.73)	0.01
**Tungsten**						
Continuous	1.75 (1.23, 2.49)	< 0.01	1.74 (1.20, 2.52)	< 0.01	1.96 (1.32, 2.92)	< 0.01
Q1	Reference		Reference		Reference	
Q2	1.17 (0.98, 1.40)	0.08	1.16 (0.97, 1.39)	0.11	1.14 (0.94, 1.39)	0.19
Q3	1.55 (1.31, 1.85)	< 0.01	1.49 (1.25, 1.78)	< 0.01	1.44 (1.19, 1.75)	< 0.01
Q4	1.60 (1.35, 1.90)	< 0.01	1.57 (1.31, 1.87)	< 0.01	1.57 (1.29, 1.90)	< 0.01
***P*** **for trend**	9.00 (4.12, 19.65)	< 0.01	8.10 (3.59, 18.29)	< 0.01	8.45 (3.46, 20.65)	< 0.01
**Uranium**						
Continuous	11.27 (2.39, 53.27)	< 0.01	9.09 (2.03, 40.63)	0.00	7.84 (1.63, 37.59)	0.01
Q1	Reference		Reference		Reference	
Q2	1.26 (1.06, 1.51)	0.01	1.20 (1.00, 1.43)	0.05	1.12 (0.92, 1.36)	0.26
Q3	1.41 (1.18, 1.67)	< 0.01	1.20 (1.01, 1.44)	0.04	1.13 (0.93, 1.38)	0.22
Q4	1.57 (1.32, 1.86)	< 0.01	1.34 (1.12, 1.60)	< 0.01	1.34 (1.10, 1.63)	< 0.01
***P*** **for trend**	3273357.52 (6473.41, inf.)	< 0.01	8803.56 (12.25, 6326095.72)	0.01	43188.09 (32.09, inf.)	< 0.01

Model 1: crude model;

Model 2: adjusted for age, sex, race, family income-poverty ratio, and education;

Model 3: adjusted for age, sex, race, family income-poverty ratio, education, smoking status, alcohol intake, serum cotinine concentration, BMI, hypertension, diabetes, physical activity and eGFR.

Abbreviation: Q: quartile

### WQS regressions to assess the associations of metals co-exposure and frailty

**Fig. 1:**
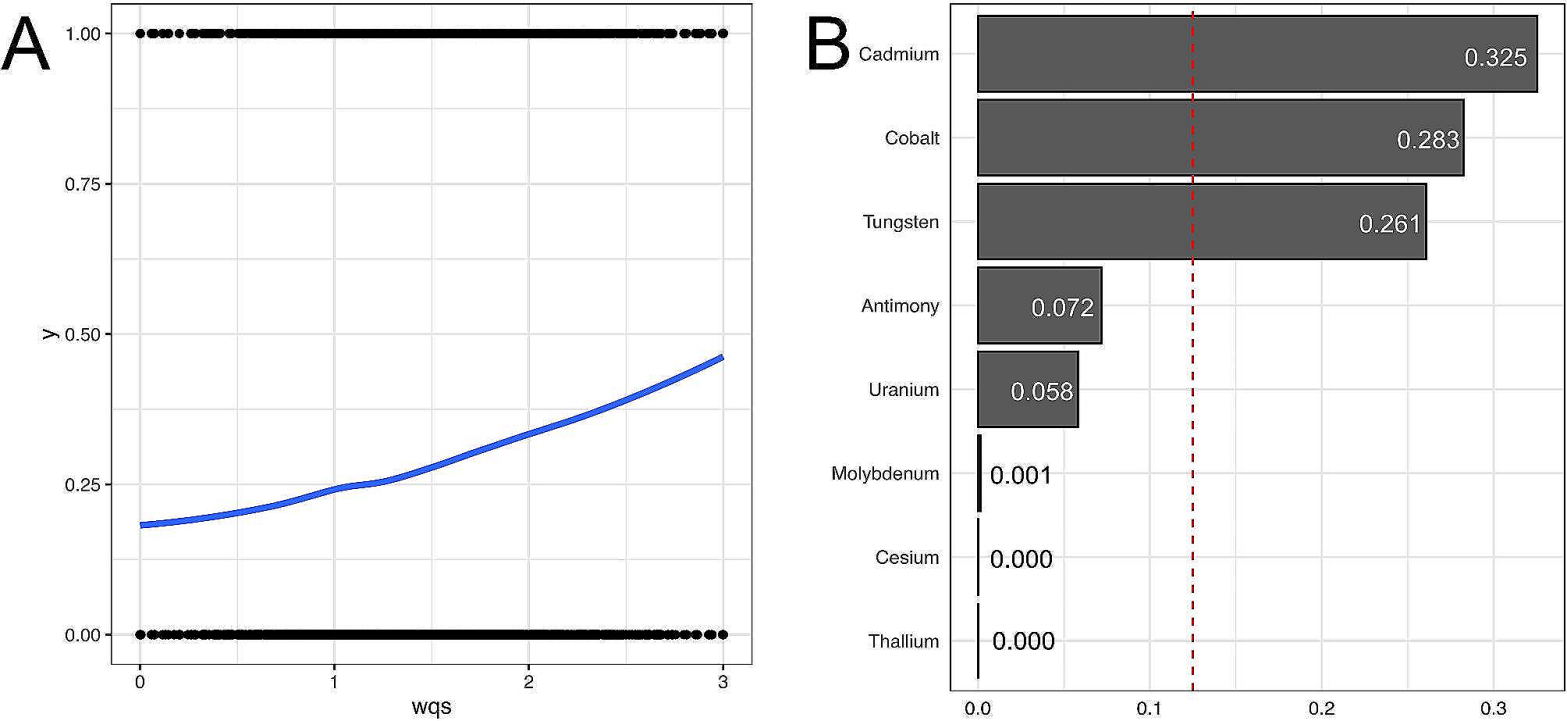
Positive associations of urinary metals with frailty risk in total population and different subgroups were estimated by WQS models. A for joint effect of mixture exposures, B for single scaled effect size of heavy metal exposure. Models were adjusted for age, sex, race, family income-poverty ratio, education, smoking status, alcohol intake, serum cotinine concentration, BMI, hypertension, diabetes, physical activity and eGFR.

We applied the WQS model to investigate the association between the combination of eight urinary heavy metals and the prevalence of frailty. As shown in Supplementary Table S3 and Fig. 1(A), the WQS index indicated that the heavy metal mixture was positively associated with the prevalence of frailty (OR = 1.67, 95% CI 1.45–1.94, *P* < 0.001). As shown in Fig. 1(B), after adjusting for all covariates, cadmium has the highest impact weight on frailty risk at 0.325, and cobalt, tungsten, antimony, and uranium have weights of 0.283, 0.261, 0.072, and 0.058, respectively, with cadmium, cobalt, and tungsten considered to be the most contributing according to thresholds set by the WQS regression. The WQS regressions in the negative direction regression did not show any significant association between heavy metal mixtures and prevalence of frailty (OR = 0.95, 95% CI 0.86–1.04, *P* = 0.263), as shown in Supplementary Table S3.

### BKMR model to assess the associations between co-exposure of metals and frailty

**Fig. 2:**
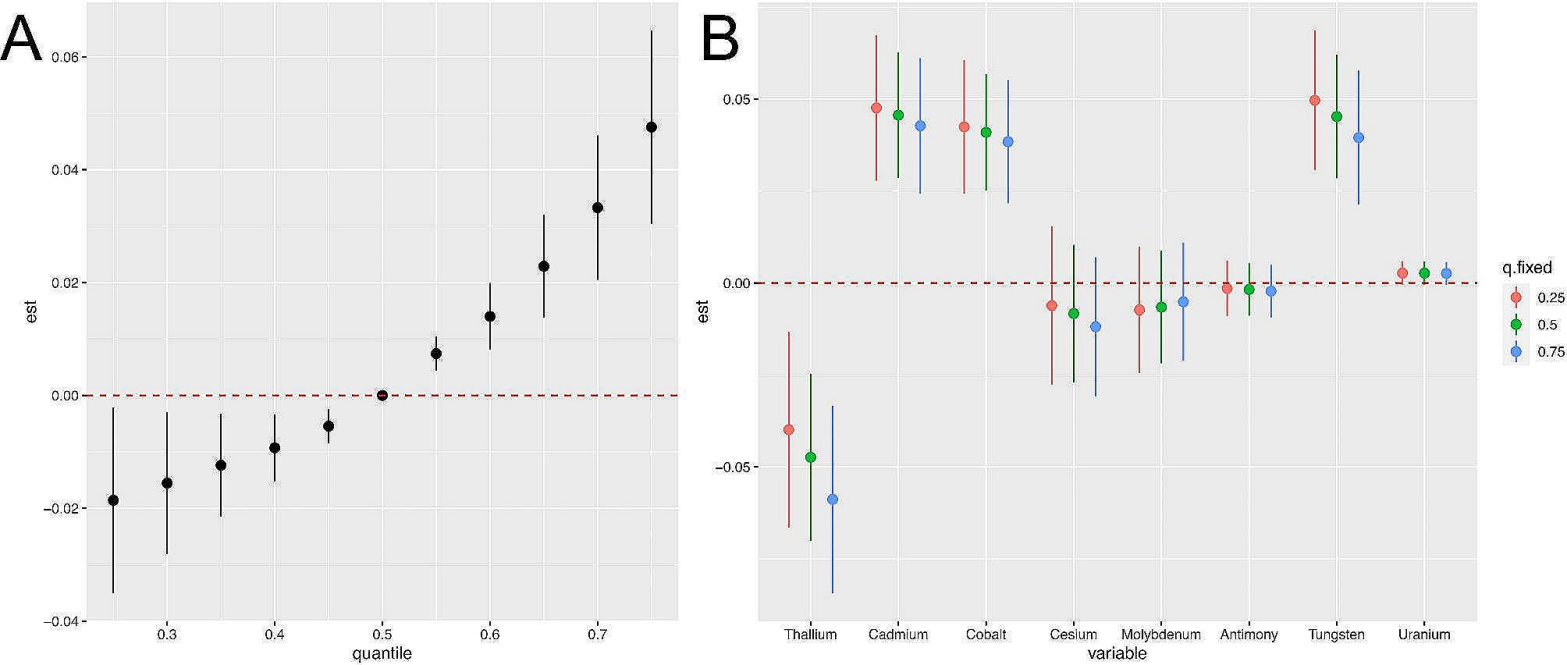
The overall effect of heavy metals on frailty estimated by BKMR models (**A**). Associations of urinary heavy metals with frailty risk when other all heavy metals were held at their corresponding 25th (red), 50th (green) or 75th (blue) percentile, respectively (**B**). Models were adjusted for age, sex, race, family income-poverty ratio, education, smoking status, alcohol intake, serum cotinine concentration, BMI, hypertension, diabetes, physical activity and eGFR

In this study, we used a BKMR model to investigate the dose-response relationship between individual heavy metal exposure and the occurrence of frailty in a mixed exposure context. In the BKMR model, the risk of frailty was significantly increased when co-exposed to a mixture of heavy metals above the 50th percentile compared to the median (Fig. 2(A)). Figure 2(B) reveals the effect of single heavy metal levels on the prevalence of frailty when controlling for the 25th, 50th, and 75th percentiles of other metals. A significant positive effect can be seen between cadmium, cobalt and tungsten exposure and frailty risk. This result is consistent with the results of the WQS regression. Supplementary Table S4 summarizes the PIP in the BKMR model. Cadmium, cobalt, and tungsten had the highest PIP values, which means that exposure to these metals can cause the greatest frailty-promoting effect. In addition, Supplementary Figure S2 shows no potential interactions between cadmium, cobalt and tungsten from the 25th to the 75th percentile.

### Relationship between frailty-promoting heavy metals in urine and mortality in frail patients

**Fig. 3:**
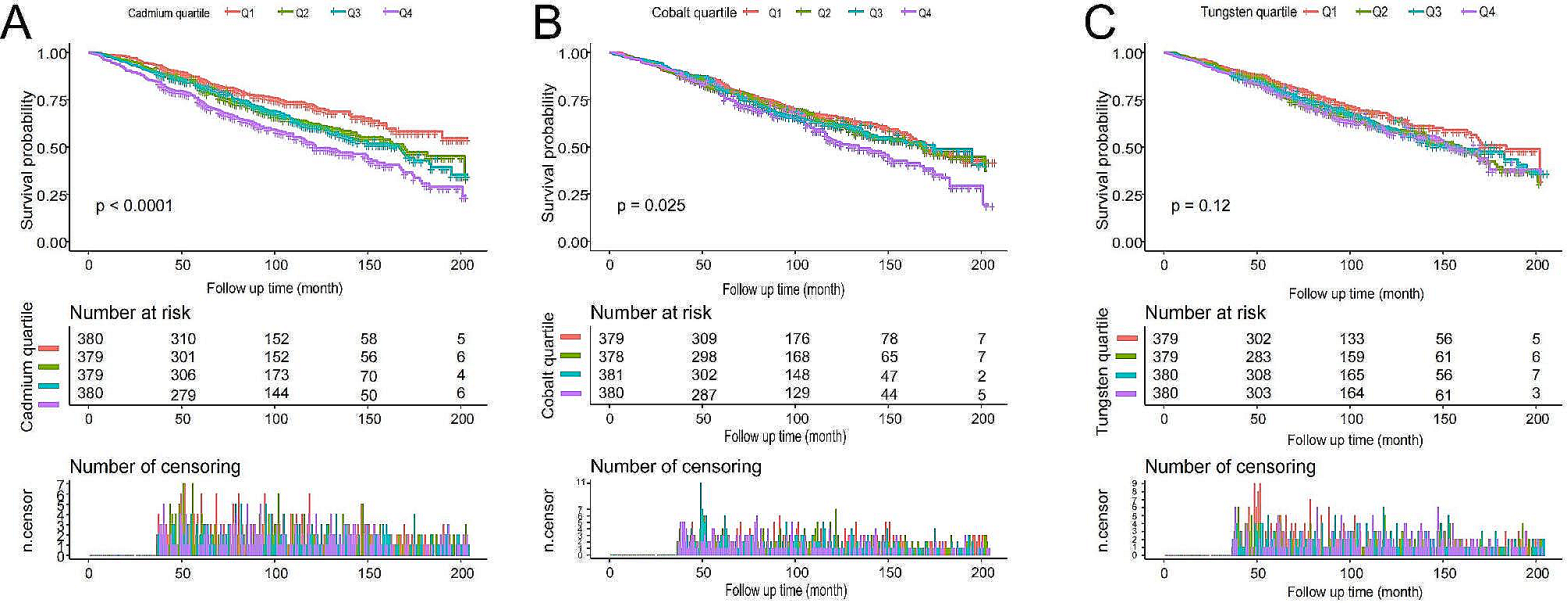
The KM survival curve of the study populations based on the cadmium (**A**), cobalt (**B**) and tungsten (**C**) group. Q: quartile

A total of 560 deaths (36.9%) were reported during a mean follow-up period of 92.4 months (range: 3-204 months). WQS regression identified cadmium, cobalt, and tungsten as the heavy metals with the most pronounced role in promoting frailty. We included these three heavy metals in the survival analysis of patients with frailty. As shown in Fig. 3(A) and (B), Kaplan-Meier analyses indicated that patients in the highest quartile of urinary cadmium and cobalt concentrations had a significantly higher risk of death (P-values < 0.001 and = 0.025, respectively). However, as shown in Fig. 3(C), KM curve analysis indicated that the association between reduced urinary tungsten and reduced mortality was not significant (*P* = 0.12).

In the unadjusted Cox regression model, increased levels of cadmium and cobalt exposure could significantly increase the risk of death with HR95% of 1.77 (1.44, 2.19) and 1.33 (1.16, 1.52), respectively. Translating urinary cadmium and cobalt exposure levels into categorical variables, the mortality rate increased progressively with increasing cadmium and cobalt exposure levels (both P for trend < 0.01). On the other hand, increasing tungsten exposure did not increase mortality in frail patients. These associations were consistent in the minimally adjusted and fully adjusted models (Table 3).

**Table 3:** HR (95% CI) in frailty associated with log-urinary heavy metals levels according to cox regression

Urine Metals (log-µg/g creatinine)	Model 1		Model 2		Model 3	
	HR (95% CI)	*P* value	HR (95% CI)	*P* value	HR (95% CI)	*P* value
**Cadmium**						
Continuous	1.77 (1.44, 2.19)	< 0.01	1.95 (1.57, 2.43)	< 0.01	1.96 (1.53, 2.52)	< 0.01
Q1	Reference		Reference		Reference	
Q2	1.38 (1.06, 1.79)	0.02	1.32 (1.02, 1.72)	0.04	1.36 (1.04, 1.77)	0.03
Q3	1.43 (1.11, 1.84)	0.01	1.44 (1.11, 1.86)	0.01	1.48 (1.13, 1.94)	< 0.01
Q4	1.97 (1.54, 2.52)	< 0.01	1.95 (1.52, 2.51)	< 0.01	1.93 (1.45, 2.56)	< 0.01
***P*** **for trend**	2.19 (1.65, 2.90)	< 0.01	2.21 (1.66, 2.95)	< 0.01	2.12 (1.53, 2.94)	< 0.01
**Cobalt**						
Continuous	1.33 (1.16, 1.52)	< 0.01	1.24 (1.08, 1.42)	< 0.01	1.30 (1.13, 1.49)	< 0.01
Q1	Reference		Reference		Reference	
Q2	1.09 (0.86, 1.38)	0.48	1.13 (0.89, 1.43)	0.32	1.29 (1.01, 1.65)	0.04
Q3	1.09 (0.86, 1.39)	0.48	1.14 (0.90, 1.46)	0.28	1.30 (1.02, 1.67)	0.04
Q4	1.40 (1.11, 1.76)	< 0.01	1.33 (1.06, 1.68)	0.02	1.57 (1.23, 2.01)	< 0.01
***P*** **for trend**	1.65 (1.18, 2.30)	< 0.01	1.50 (1.07, 2.09)	0.02	1.81 (1.29, 2.56)	< 0.01
**Tungsten**						
Continuous	1.25 (0.72, 2.16)	0.43	1.28 (0.75, 2.19)	0.36	1.23 (0.71, 2.13)	0.45
Q1	Reference		Reference		Reference	
Q2	1.29 (1.01, 1.65)	0.04	1.25 (0.98, 1.60)	0.08	1.20 (0.94, 1.54)	0.15
Q3	1.22 (0.96, 1.57)	0.11	1.22 (0.95, 1.56)	0.12	1.25 (0.97, 1.61)	0.08
Q4	1.31 (1.03, 1.67)	0.03	1.34 (1.05, 1.72)	0.02	1.30 (1.01, 1.66)	0.04
***P*** **for trend**	2.18 (0.84, 5.62)	0.11	2.60 (0.99, 6.80)	0.05	2.29 (0.87, 6.05)	0.09

Model 1: crude model

Model 2: adjusted for age, sex, race, family income-poverty ratio, and education

Model 3: adjusted for age, sex, race, family income-poverty ratio, education, smoking status, alcohol intake, serum cotinine concentration, BMI, hypertension, diabetes, physical activity and eGFR

Abbreviation: Q: quartile

## Discussion

In the present research, we scrutinized the association between exposure to multiple heavy metals and the risk of frailty, alongside the long-term survival rates of frail patients within a middle-aged and elderly demographic, utilizing a significant population sample. Our analysis employed various statistical methods, including logistic regression, Cox regression, WQS regression, and BKMR model. The results unequivocally indicate a notable positive correlation between mixed heavy metal exposure and the risk of frailty. Particularly, cadmium, cobalt, and tungsten were identified as significant contributors to an increased frailty risk. Furthermore, exposure to cadmium and cobalt significantly worsened the long-term outcomes for frail patients. These insights underscore the urgent need for preventive measures against exposure to these heavy metals -- cadmium, cobalt, and tungsten in particular -- as robust safeguards for middle-aged and elderly individuals to reduce the risk of frailty and enhance long-term health prospects.

Frailty is characterized as a complex syndrome of crucial importance in geriatric medicine [39]. It manifests through a significant reduction in physical and mental functionality and reserve capacity. This decline severely impairs a patient’s capacity to endure external stress, which profoundly affects their overall health and life expectancy [40]. Previous studies have primarily focused only on the effects of single exposures to lead and cadmium on the risk of frailty [16, 17, 41]. This study’s distinct approach involved analyzing concomitant exposures and employing an extended follow-up period to observe frail patients. he findings confirm that exposure to multiple heavy metals, as threatening external stressors, significantly accelerates the onset of frailty. Moreover, this exposure directly deteriorates the long-term prognosis for these patients.

In the present study, cadmium, cobalt and tungsten emerged as the heavy metals exhibiting the most significant impact on frailty. Cadmium, regarded as one of the most toxic and widely distributed heavy metals, tends to accumulate in the liver [42], kidneys [43], and bones [44], which leads to a close association with renal tubular damage, urinary calcium loss, accelerated bone demineralization, and the development of osteoporosis [45]. Moreover, cadmium has been linked in the pathogenesis of diabetes, hypertension, and an elevated risk of cardiovascular disease, posing a serious threat to population health through various pathways [46]. The primary dietary sources of cadmium, including leafy vegetables, grains, nuts, and organ meats, reflect the wide range of potential exposure pathways, compounded by the significant contribution of cigarette smoke in non-occupational settings [47]. Industrial activities further increase the risk, particularly in battery manufacturing, metal plating, and certain types of welding and soldering, necessitating stringent workplace safety standards [48, 49]. The WHO’s recommendation of a tolerable weekly intake of 5.8 µg/kg body weight underscores the importance of minimizing exposure to mitigate health risks [48].

Tungsten exhibits considerable immunotoxic, pulmonary, and carcinogenic properties [50], which are largely attributed to mechanisms involving direct DNA damage, the generation of reactive oxygen species (ROS), and epigenetic modulation [51]. Furthermore, tungsten possesses the capacity to enhance the impacts of co-exposures, stimulants, stressors, and cellular processes, potentially exacerbating toxicity or leading to more severe pathological alterations [52]. Tungsten, primarily encountered in occupational settings [51], such as in the production of hard metals and electronics, and to a lesser extent through environmental exposure, is devoid of established intake recommendations [50]. The absence of a designated RDI or Upper Intake Level (UIL) for tungsten underscores the need for further research to evaluate its health impacts fully and establish guidelines for safe exposure levels.

The potential health risks associated with cobalt exposure have garnered significant attention, particularly in the context of its utilization in orthopedic implants [53]. In humans, the effects of ionized cobalt are primarily mediated through mechanisms involving ROS production, lipid peroxidation, mitochondrial dysfunction, disturbances in the homeostasis of calcium and iron, interactions with the body’s feedback system affecting erythropoiesis [54], interference with thyroid iodine uptake, the induction of genotoxic effects, and disruption of DNA repair processes [55]. However, cobalt, despite its potential toxicity in excessive amounts, plays a crucial role in human health as a component of vitamin B12 [56]. The dietary intake of cobalt, chiefly through meat, fish, dairy products, and eggs, contributes to its essential physiological functions, notably red blood cell formation [57]. However, the absence of a specific Recommended Dietary Intake (RDI) for cobalt, with guidelines provided indirectly through vitamin B12 recommendations (approximately 2.4 µg for adults), underscores the delicate balance required to ensure adequate intake for health optimization while preventing toxic exposure [55, 58].

Molybdenum’s recognition as an essential trace element [59], with delineated dietary sources and firm intake recommendations (45 µg per day for adults, with an Upper Intake Level of 2,000 µg or 2 mg), exemplifies effective management of trace element exposure [60]. Dietary sources, including legumes, grains, leafy vegetables, liver, and milk, facilitate the necessary intake for its role in enzymatic processes, while the established guidelines ensure both adequacy and protection against toxicity [61].

The contrasting profiles of these trace elements—ranging from the essential nutrients cobalt and molybdenum to the toxic metals cadmium and tungsten—illustrate the critical challenge in environmental health and nutritional science [62]. It involves ensuring adequate intake of essential nutrients while preventing overexposure to harmful substances. This balance is essential for public health initiatives aimed at reducing exposure to toxic metals and ensuring sufficient levels of essential nutrients, indicating the need for an integrated approach that considers both dietary intake and environmental exposure [61].

The presence of heavy metals, which may originate from both environmental and non-environmental sources, can contribute to interactive effects on health outcomes, including frailty. Recognizing the multifaceted sources of these metals, our study employed three distinct statistical models to explore the association between heavy metal exposure and frailty comprehensively [24]. Binary logistic regression, while offering straightforward and interpretable results, encounters limitations in estimating effects of compounds that exhibit high inter-correlations [23]. To address the complexities inherent in analyzing the relationship between mixed exposures to heavy metals and health outcomes, we adopted two widely recognized statistical methodologies: WQS regression [33] and BKMR models [20]. Analyses leveraging both models consistently identified significant positive correlations between exposure to mixtures of metals and the risk of frailty, pinpointing cadmium, cobalt, and tungsten as having the most pronounced pro-frailty effects. The prognostic analyses focusing on these metals highlight the extended prognostic hazards associated with exposure to cadmium and cobalt, underlining the importance of considering both environmental and non-environmental sources in assessing the health risks of heavy metal exposure.

The present study possesses several notable strengths. Firstly, it represents a pioneering investigation that explores the combined impact of multiple heavy metal exposures on frailty among middle-aged and older adults [33]. This innovative approach highlights the need for further dedicated research to explore the environmental determinants of frailty. In addition, this study used various reliable statistical models [33], a large sample size, and a long follow-up period to enhance the reliability of the findings. Moreover, the study employs a health deficit-based frailty index assessment, a widely recognized and valid method [63].

It is crucial to acknowledge and address the inherent limitations of this study. The present study utilized a statistical strategy of cross-sectional study to explore the association between heavy metal exposure and frailty incidence, which may have resulted in failure to establish a causal association. Further future cohort studies are essential in order to validate the identified associations and strengthen the findings. Additionally, it is important to note that this investigation solely focuses on the United States population, which raises questions about the generalizability of the results to other national and regional contexts. Lastly, despite the rigorous methodological approach employed in this study, the potential impact of unmeasured confounding variables on the observed outcomes remains unresolved and necessitates further investigation.

## Conclusions

In conclusion, our study analyzed a large cohort sample from the United States and revealed a significant positive correlation between heavy metal exposure mixtures and the incidence of frailty in middle-aged and older adults and mortality in frail patients. Among the heavy metal exposure mixtures, cobalt, cadmium, and tungsten were considered as the most influential heavy metals in causing frailty. Of these, cobalt and cadmium have been shown in prognostic analyses to further directly affect the long-term life expectancy of frail patients. This study emphasizes the critical role of modifiable environmental exposures in the prevention, management and intervention of frailty in middle-aged and older adults, leading to improved health status and long-term prognosis.

### Electronic supplementary material

Below is the link to the electronic supplementary material.

Supplementary Material 1

## Data Availability

No datasets were generated or analysed during the current study.
