# Supplementary material for: The association of urinary heavy metal exposure with frailty susceptibility and mortality in middle-aged and older adults: a population-based study
Source: Arch Public Health. 2024 Mar 27;82:44. doi: 10.1186/s13690-024-01275-8 (PMC10967095; doi:10.1186/s13690-024-01275-8)

**Supplementary Material**

**The Association of Urinary Heavy Metal Exposure with Frailty Susceptibility and Mortality in Middle-aged and Older Adults: A Population-Based Study**

**Supplementary Table S1**. Variables in the 49-Item frailty index and their respective scorings

| Variable | Scoring | | |
| --- | --- | --- | --- |
| Cognition | Yes=1, No=0 |  |  |
| 1. Experience confusion/memory problems | Difficulty=1, No Difficulty=0 |  |  |
| Dependence | Difficulty=1, No Difficulty=0 |  |  |
| 2. Managing money | Difficulty=1, No Difficulty=0 |  |  |
| 3. Stooping, crouching, kneeling | Difficulty=1, No Difficulty=0 |  |  |
| 4. Lifting or carrying | Difficulty=1, No Difficulty=0 |  |  |
| 5. House chore | Difficulty=1, No Difficulty=0 |  |  |
| 6. Preparing meals | Difficulty=1, No Difficulty=0 |  |  |
| 7. Standing up from armless chair | Difficulty=1, No Difficulty=0 |  |  |
| 8. Getting in and out of bed difficulty | Difficulty=1, No Difficulty=0 |  |  |
| 9. Using fork, knife, drinking from cup | Difficulty=1, No Difficulty=0 |  |  |
| 10. Dressing yourself | Difficulty=1, No Difficulty=0 |  |  |
| 11. Standing for long periods difficulty | Difficulty=1, No Difficulty=0 |  |  |
| 12. Grasp/holding small objects | Difficulty=1, No Difficulty=0 |  |  |
| 13. Attending social event | Difficulty=1, No Difficulty=0 |  |  |
| 14. Push or pull large objects | Difficulty=1, No Difficulty=0 |  |  |
| 15. Walking for a quarter mile difficulty | Difficulty=1, No Difficulty=0 |  |  |
| 16. Walking up 10 steps difficulty | Difficulty=1, No Difficulty=0 |  |  |
| Depressive Symptoms |  |  |  |
| 17. Have little interest in doing things | Nearly every day=1, More than half the days=0.66, Several days=0.33, Not at all=0 |  |  |
| 18. Feeling down, depressed, or hopeless | Nearly every day=1, More than half the days=0.66, Several days=0.33, Not at all=0 |  |  |
| 19. Trouble sleeping or sleeping too much | Nearly every day=1, More than half the days=0.66, Several days=0.33, Not at all=0 |  |  |
| 20. Feeling tired or having little energ | Nearly every day=1, More than half the days=0.66, Several days=0.33, Not at all=0 |  |  |
| 21. Poor appetite or overeating | Nearly every day=1, More than half the days=0.66, Several days=0.33, Not at all=0 |  |  |
| 22. Feeling bad about yourself | Nearly every day=1, More than half the days=0.66, Several days=0.33, Not at all=0 |  |  |
| 23. Trouble concentrating on things | Nearly every day=1, More than half the days=0.66, Several days=0.33, Not at all=0 |  |  |
| Comorbidities |  |  |  |
| 24. Arthritis | Yes=1, Suspect=0.5, No=0 |  |  |
| 25. Thyroid problems | Yes=1, Suspect=0.5, No=0 |  |  |
| 26. Chronic bronchitis | Yes=1, Suspect=0.5, No=0 |  |  |
| 27. Cancer | Yes=1, Suspect=0.5, No=0 |  |  |
| 28. Congestive heart failure | Yes=1, Suspect=0.5, No=0 |  |  |
| 29. Coronary heart disease | Yes=1, Suspect=0.5, No=0 |  |  |
| 30. Angina | Yes=1, Suspect=0.5, No=0 |  |  |
| 31. Heart attack | Yes=1, Suspect=0.5, No=0 |  |  |
| 32. Stroke | Yes=1, Suspect=0.5, No=0 |  |  |
| 33. Blood pressure | Yes=1, Suspect=0.5, No=0 |  |  |
| 34. Diabetes | Yes=1, Suspect=0.5, No=0 |  |  |
| 35. weak/failing kidneys | Yes=1, Suspect=0.5, No=0 |  |  |
| 36. Urinary Leakage | Yes=1, Suspect=0.5, No=0 |  |  |
| Hospital Utilization and Access to Care |  |  |  |
| 37. Self-rated health | Fair, poor=1, Excellent, Very good, good=0 |  |  |
| 38. Health now compared with 1 year ago | Worse=1, About the same, better=0 |  |  |
| 39. Overnight hospital patient in past year | Yes=1, No=0 |  |  |
| 40. Frequency of health care use during past year | None=0, 1-5=0.5, More than 5=1 |  |  |
| 41. Number of prescribed medications | None=0, 1-4=0.5, 5 and more=1 |  |  |
| Physical Performance and Anthropometry |  |  |  |
| 42. Body mass index | <18.5, ≥30=1 |  |  |
|  | 25-<30=0.5 |  |  |
|  | 18.5-25=0 |  |  |
| 43. Handgrip strength | MALE: |  | FAMELE: |
|  | For BMI≤24，GS≤29 |  | For BMI≤23，GS≤17 |
|  | For BMI24.1-28, GS≤30 |  | For BMI23.1-26,GS≤17.3 |
|  | For BMI＞28, GS≤32=1 |  | For BMI26.1-29,GS＜18 |
|  |  |  | For BMI＞29,GS≤21=1 |
| Laboratory Values |  |  |  |
| 44. Glycohemoglobin (%) | 0%-5.7%=0, >5.7%=1 |  |  |
| 45. Red blood cell count (million cells/mL) | M: 4.7-6.1=0, Other=1 |  | F: 4.2-5.4=0, Other=1 |
| 46. Hemoglobin (g/dL) | M: 13.5-18=0, Other=1 |  | F: 12-16=0, Other=1 |
| 47. Red cell distribution width (%) | 11.6-14.6=0, Other=1 |  |  |
| 48. Lymphocyte percent (%) | 20-40=0,Other=1 |  |  |
| 49. Segmented neutrophils percent (%) | 40-80=0,Other=1 |  | |

**Supplementary Table S2**. Detection rates of 8 urinary metals

| Heavy metals | Detection rates | LODs(ug/L) |
| --- | --- | --- |
| Cadmium | 0.847 | 0.036 |
| Cobalt | 0.992 | 0.023 |
| Cesium | 1.000 | 0.086 |
| Molybdenum | 1.000 | 0.800 |
| Antimony | 0.754 | 0.022 |
| Thallium | 0.995 | 0.018 |
| Tungsten | 0.883 | 0.018 |
| Uranium | 0.839 | 0.002 |

LOD, limit of detection.

**Supplementary Table S3**. The joint effect of heavy metals mixtures on the prevalence of frailty in WQS model in positive and negative direction.

|  | **OR(95%CI)** | **P-value** |
| --- | --- | --- |
| Positive |  |  |
| WQS index | 1.67 (1.45,1.94) | <0.001 |
| Negative |  |  |
| WQS index | 0.95 (0.86,1.04) | 0.263 |

WQS: weighted quantile sum; CI: confidence interval; Models were adjusted for all covariates.

**Supplementary Table S4** PIPs of each heavy metal for the prevalence of frailty in BKMR model.

| Variable | PIP |
| --- | --- |
| Thallium | 0.9892 |
| Cadmium | 1 |
| Cobalt | 1 |
| Cesium | 0.1422 |
| Molybdenum | 0.0462 |
| Antimony | 0.3418 |
| Tungsten | 1 |
| Uranium | 0.7728 |

This model adjusted for all covariates. PIP, posterior inclusion probability; BKMR, Bayesian kernel machine regression

**Supplementary Figure S1**. Flow Diagram of Inclusion/Exclusion Criteria.


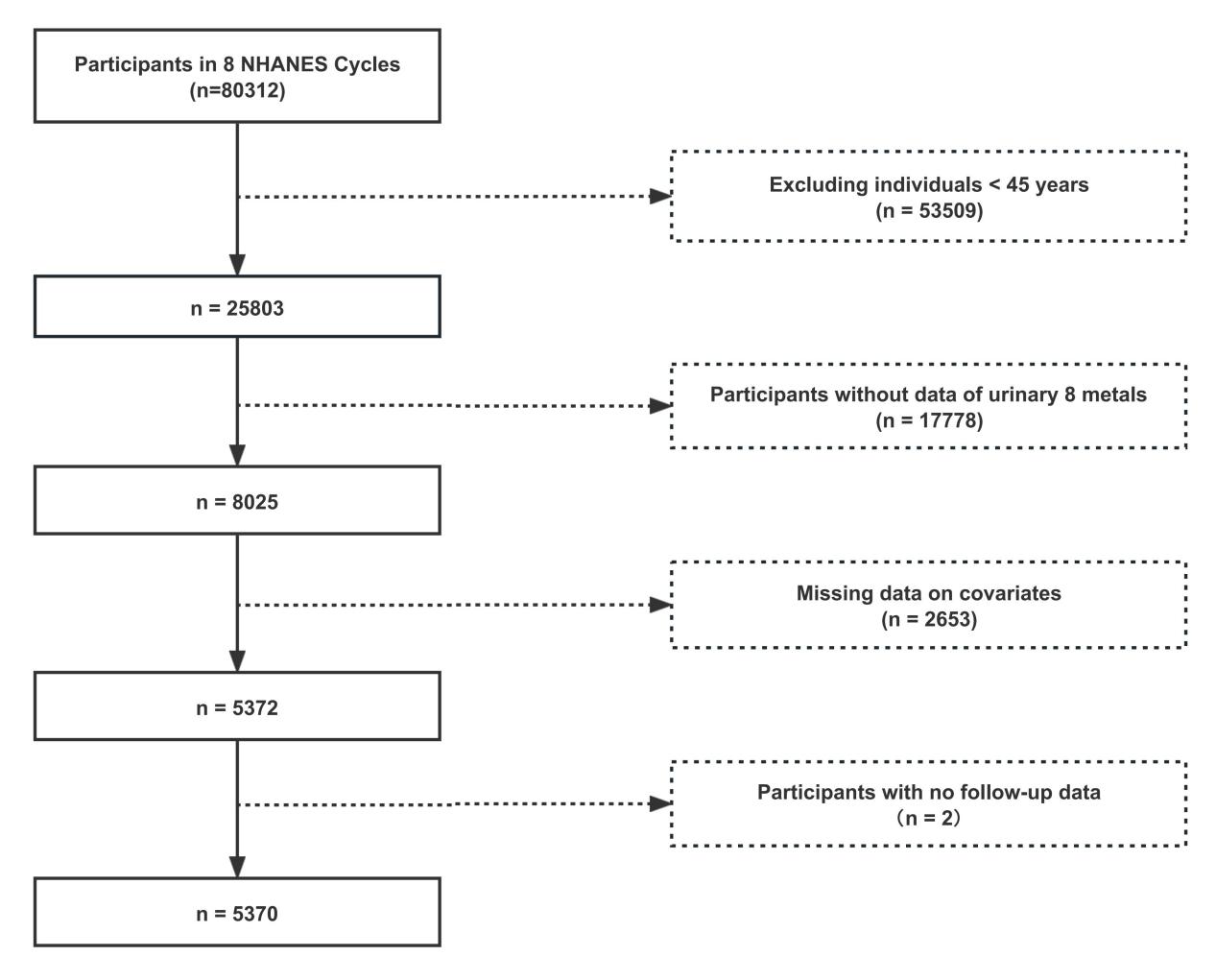


**Supplementary Figure S2**. Bivariate exposure–response functions for the when another pollutant was fixed at varying (25th, 50th, 75th) percentiles and other pollutant was fixed at the median estimated by BKMR models. Models were adjusted for age, sex, race, family income-poverty ratio, education, smoking status, alcohol intake, serum cotinine concentration, urine creatitine, BMI, hypertension, diabetes, physical activity and eGFR.


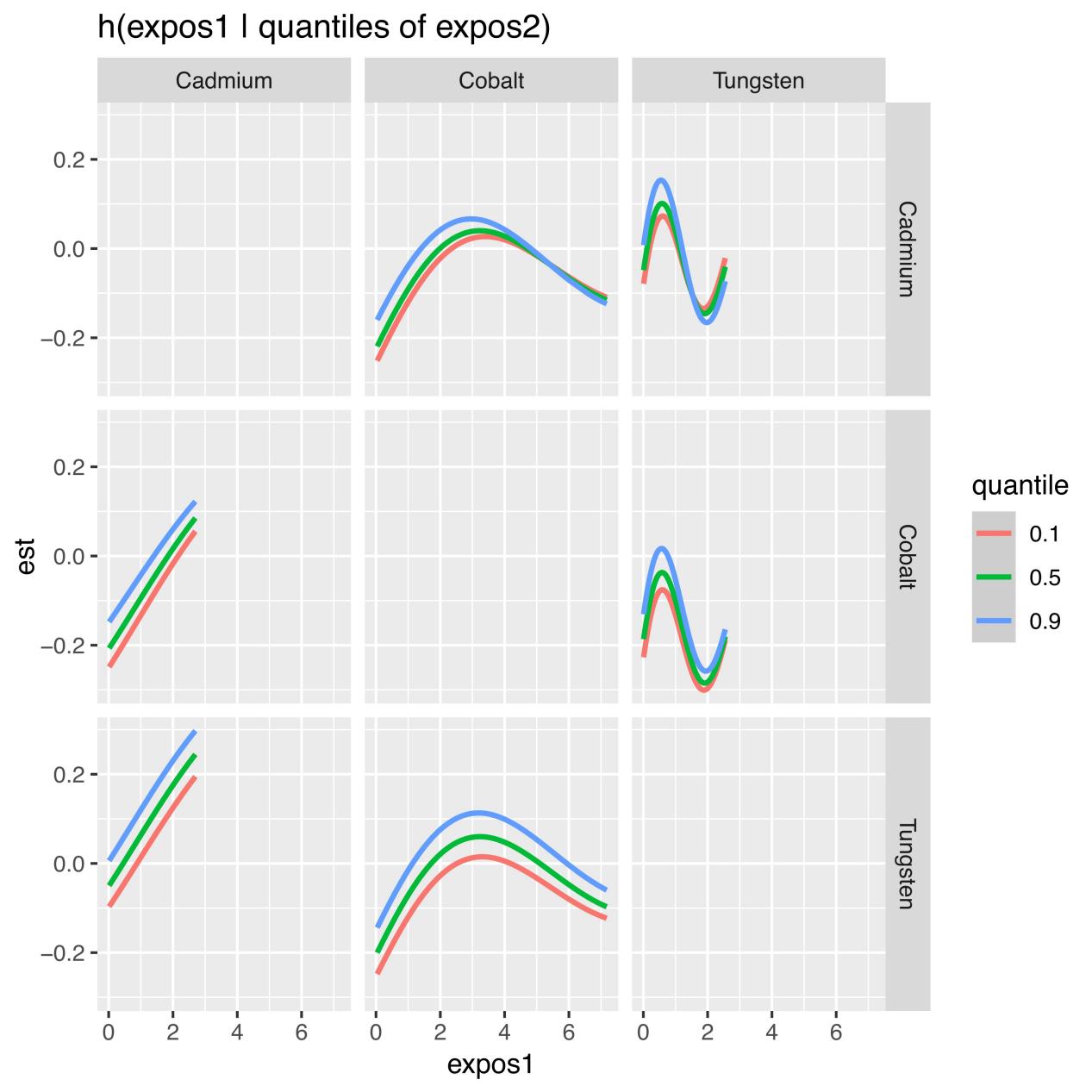

Supplement: Supplementary file 1 — Supplementary Material 1 [file 13690_2024_1275_MOESM1_ESM.docx]
